# “Drunk People Are on a Different Level”: A Qualitative Study of Reflections From Students About Transitioning and Adapting to United Kingdom University as a Person Who Drinks Little or No Alcohol

**DOI:** 10.3389/fpsyg.2021.702662

**Published:** 2022-01-27

**Authors:** Elspeth Cook, E. Bethan Davies, Katy A. Jones

**Affiliations:** ^1^School of Medicine, University of Nottingham, Nottingham, United Kingdom; ^2^NIHR MindTech MedTech Co-operative, Institute of Mental Health, School of Medicine, University of Nottingham, Nottingham, United Kingdom; ^3^Academic Unit of Mental Health and Clinical Neurosciences, School of Medicine, University of Nottingham, Nottingham, United Kingdom

**Keywords:** alcohol, sober, students, social, well-being, university

## Abstract

**Background:**

Though sobriety in young people is on the rise, students who drink little or no alcohol may experience social exclusion at University, impacting well-being. We aim to understand the social experiences of United Kingdom (UK) undergraduate students who drink little or no alcohol.

**Methods:**

A mixed-methods study using semi-structured, one-to-one interviews and the 24-Item Social Provisions Scale and Flourishing Scale with 15 undergraduate students who drink little or no alcohol. Descriptive statistics are presented for quantitative data and thematic analysis for qualitative.

**Results:**

Eight main themes and four subthemes were generated from thematic analysis summarised in two sections ‘views of drinkers from non-drinkers’ and ‘how peer pressure feels and how people deal with it.’ The initial transition to University represented a challenge, where participants struggled to find their ‘true’ friends. However, students generally had high levels of social provision, well-being and enjoyed close friendships with fewer casual acquaintances. All students experienced some kind of peer pressure (of a varying extremity) and developed coping strategies when in social situations involving alcohol. Fear of missing out on the ‘typical’ University experience heightened self-imposed expectations to drink. Despite participants acknowledging their counter-normative behaviour, some felt they were subject to stigmatisation by drinkers, doubting their non-drinker status, causing feelings of exclusion or being ‘boring.’ Their desire to ‘be like everyone else’ exposed some insight into the negative stereotypes of sobriety, including frustration behind alcohol’s status elevation.

**Conclusion:**

Students adopt strategies to minimise peer pressure and to fit in. Future research should interrogate drinkers’ perceptions of their sober peers to deepen understanding, better break down ‘us and them,’ and mitigate future expectations within the University drinking culture.

## Introduction

During the transition to and through university, students are faced with the challenging task of forming new relationships in unfamiliar situations (Pittman and Richmond, 2008). At the same time, they are inducted into university cultural norms which include drinking alcohol. A survey conducted by the National Union of Students Press Team of 2,215 United Kingdom (UK) students showed 20% get drunk ‘on purpose’ once a week and 79% said drinking and getting drunk was part of the University experience^1^. Studies exploring drinking practices among university students describe alcohol’s effect as a ‘gateway’ or ‘emollient’ into social networks (Griffin et al., 2009; Conroy and de Visser, 2015). For example, Ireland’s (2019) qualitative study of first-year United Kingdom university students demonstrated alcohol’s importance during Fresher’s week, showing its role in reducing initial inhibition and assisting peer bonding (Baron and Kerr, 2003; Fuller et al., 2018). Drinking alcohol is associated with ‘having fun,’ and ‘socialising’ (Park, 2004; Lee et al., 2011) and according to the aforementioned survey^1^, 70% of students believe they must drink to fit in.

Despite student views, consumption trends show ‘teetotalism’ or ‘sobriety’ (defined as drinking no alcohol at all) has risen by 40% for 16--24-year-olds^2^ with 21% of students in the aforementioned survey choosing not to drink. This change has most likely arisen from general societal changes such as stricter parental attitudes, different leisure activities, and an expanding multicultural society (Herring et al., 2014; Fat et al., 2018). Kraus et al. (2020) hypothesise a generational shift, where the cultural politics of drinking at the time of a cohort’s young adulthood predicts their consumption in later life. Qualitative studies have explored additional reasons for not drinking. These include sporting commitments, a personal or family history of alcohol misuse, not liking the taste or effect (Nairn et al., 2006; Piacentini and Banister, 2009), or negative consequences of drinking (e.g., impaired academic performance; Mustaine and Tewksbury, 2005).

Having genuine social support and good quality friends is associated with better well-being at University (Buote et al., 2007; Demir and Davidson, 2013). Some research has explored peer networks and the interpersonal well-being of students and young people who do not drink alcohol. On one hand, Conroy and de Visser (2018) suggest these students have more opportunities to develop an ‘inclusive and fulfilling social network.’ Indeed, Herring et al. (2014) argue it is drinkers who limit their social abilities by using alcohol as a social ‘crutch.’ Thus, while sobriety may be the more challenging path, it may be enriching for individual growth. On the other hand, the ‘unsociable’ stigma of not drinking may automatically devalue a person’s social status (Herman-Kinney and Kinney, 2013), depriving people access to meaningful social situations (Conroy and de Visser, 2014). Non-drinkers do report taking longer to form their peer groups than their drinking peers (Brown and Murphy, 2020). Although contradictory literature suggests these students, although excluded from some social groups, can be more socially competent in others (Conroy and de Visser, 2013).

Despite more students choosing sobriety, many are subjected to overt or covert peer pressure to drink, some facing social exclusion if they do not conform (Nairn et al., 2006; Piacentini and Banister, 2009; Conroy and de Visser, 2015). A study showed 20% of students were teased directly to their faces, while 16% were called negative names (Herman-Kinney and Kinney, 2013). Heavy-drinking students in New Zealand were labelled by terms related to popularity and respect, such as a ‘social climber’ and having a ‘liver of steel,’ but also negative labels such as ‘liability’ (Robertson and Tustin, 2018). Social pressure is expressed and experienced differently depending on stereotypical expectations of gender. For example, men report pressure to binge drink to maintain their ‘masculine status’ (West and Zimmerman, 1987), being told by peers to ‘man-up’ (De Visser et al., 2009) and called a ‘weirdo’ if they do not conform (Robertson and Tustin, 2018). However, some research suggests such pressure comes from casual acquaintances rather than close friends (Supski and Lindsay, 2017).

To cope, some students develop strategies to justify or hide their decision (Nairn et al., 2006). For example, lying or providing medical reasons (Jacobs et al., 2018), adopting alternative roles such as ‘athlete’ (Herman-Kinney and Kinney, 2013), or projecting negative assumptions on to other abstainers to legitimise their position of ‘not being that type of non-drinker’ (Banister et al., 2019). ‘Coming out’ as a non-drinker is approached with caution for fear of being considered an outsider (Conroy and de Visser, 2014). For example, a qualitative study at an American university found that only 29% of students disclosed their non-drinking status to peers, however, this changed through the university years, with first-year non-drinkers being the most likely to completely conceal their identity to avoid social rejection (Herman-Kinney and Kinney, 2013).

There have been a handful of previous qualitative studies aiming to understand the experiences of young people who drink little or no alcohol. Three United Kingdom-based studies have focused specifically on the use of alcohol, including reasons for doing (or not doing) so at University (e.g., Conroy and de Visser, 2014; Jacobs et al., 2018; Banister et al., 2019). Little research has specifically examined areas of social integration and bonding at University. Where they have, such studies only investigated the experience of alcohol abstinence within the context of sports societies (Zhou and Heim, 2016), or focused exclusively on the experiences of women (Jacobs et al., 2018). Similar to the present research, Ireland (2019) explored the experiences of transitioning into university as a non-drinker by interviewing eight students in their first year at University. This study was informative, but students may not have had the time to adequately reflect on their social experiences which are likely to change over time. We hope to speak to people who are in different stages of their journey through university and will have different types of reflections on their experiences.

Few studies have explored the social experiences and well-being of a range of undergraduate University students who drink little or no alcohol. This study aims to explore their transitions, friendships, and social experiences. Their experiences will be further contextualised by self-reported levels of flourishing and perceived social support.

The study will address the following primary research questions with undergraduate University students in the United Kingdom who drink little or no alcohol:

(1) What are students reflections on how they transitioned to university and adapted to university life as a person who drinks or no alcohol?
(2) What are these students self-reported mental well-being (flourishing) and social inclusion scores?

More generic questions will be explored in this study as they add important context to people’s stories (e.g., ‘reasons for drinking little or no alcohol’). However, as these have been reported reasonably robustly in previous qualitative literature, we will focus the results reporting on more novel questions.

## Materials and Methods

### Ethical Approval and Considerations

This study was reviewed and approved by the University of Nottingham’s Division of Psychiatry and Applied Psychology Research Ethics Committee (reference number: 1663). Information was provided to participants online (via JISC online surveys). Informed consent was given online. Participants were asked if they agreed to a series of standard consent statements and to click yes to consent to the study (which took them to the next page). Those who clicked no were automatically redirected to the debriefing page. Consent was also re-confirmed verbally before the interview. Participants were informed they could disclose as much or as little information as they felt comfortable, and the interview could be stopped at any time. All participants were signposted to appropriate services if required *via* a debriefing sheet. All study data were stored securely as per the University of Nottingham (UoN)’s standard data management procedures.

The Consolidated Criteria for Reporting Qualitative Research (COREQ) Checklist was followed for reporting qualitative studies to ensure rigor in reporting qualitative research (Tong et al., 2007; Supplementary Material 1).

### Participants and Recruitment

**FIGURE 1:**
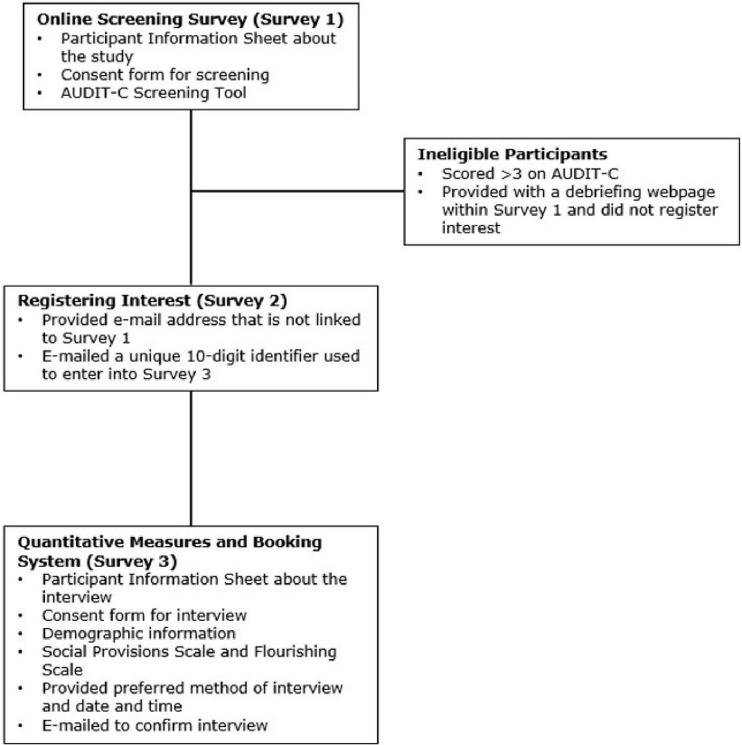
Procedural flow of participants through the study.

See Figure 1 for participant flow through the study, including eligibility screening. Target participants were current undergraduate students at the UoN who identified as drinking little or no alcohol. Only UoN students were targeted for recruitment in this study due to the short timeframe to collect the data. To be eligible, students were required to score below three on the Alcohol Use Disorders Identification Test Consumption (AUDIT-C, Bush et al., 1998) delivered by survey one. The AUDIT-C was selected because it is a quick way to identify a person’s alcohol consumption and risk of alcohol harm (Public Health England, 2020), it has also been validated in a student population (Campbell and Maisto, 2018). The highest score of three on the AUDIT-C was equivalent to a student who drank monthly or less, consumed between 1 and 4 units if they did drink and reported ‘binge’ drinking either never or monthly or less. Those who were not eligible were directed to the debriefing page and were informed they would not be offered an interview. Further, only students in their second year of University or above were eligible to take part as the study took place in the autumn semester (October–December 2020), and it was felt that first-year students had not spent that long at University and were having limited social experiences due to the COVID-19 pandemic.

Participants were a convenience sample recruited *via* online advertisements on relevant social media pages (e.g., medical student forums and University of Nottingham Student’s Union pages on Facebook) and *via* snowballing (asking participants to share the information sheet with their peers). Purposive sampling was not used, it was hoped people with a range of experiences and characteristics would volunteer. Advertisements were posted online after permission was obtained from identified gatekeepers (e.g., Student’s Union Officers and society presidents). Advertisements contained the first author’s (EC) e-mail address, a colourful graphic, and a link to the screening survey (hosted on JISC Online Surveys).

### Design

A predominately qualitative design *via* semi-structured, remote (video-call or telephone) one-to-one interviews and two quantitative measures of social inclusion and well-being *via* an online survey. Remote delivery of this research was due to United Kingdom COVID-19 restrictions. Similar research studies have used one-to-one interviews (e.g., Conroy and de Visser, 2014), and a semi-structured approach allowed for a sufficient depth of understanding and would allow participants to disclose their feelings and unique personal experiences (Braun and Clarke, 2013).

### Social Support: Social Provisions Scale-24 (SPS-24; Cutrona and Russell, 1987)

The SPS-24 was used to measure perceived social support and adaptation to stress (Cutrona and Russell, 1987; Steigen and Bergh, 2019). It was chosen as it provided an overall summary of a person’s level of perceived social provision, but also detail about key areas of interest *via* six subscales guidance (‘there is someone I could talk to about important decisions in my life’), reassurance of worth (‘I have relationships where my competence and skill are recognised’), social integration (‘There are people who enjoy the same activities that I do’), attachment (‘I have close relationships that provide me with a sense of emotional security and well-being’), nurturance (‘there are people who depend on me for help’), and reliable alliance (‘there are people I can depend on to help me if I really need it’). Each subscale comprises two positively and two negatively formed statements, creating 24 items. Participants select a number between one and four depending on the extent to which they agree with the statement (1 = ‘strongly disagree,’ 4 = ‘strongly agree’). The six-factor scores range from 4 to 16 and total scores range from 24 to 96. Negatively worded items were reverse scored. Higher scores indicate better-perceived support. Scores correlate significantly with a person’s satisfaction of their life and symptoms of depression (Cutrona and Russell, 1987).

### Well-Being: Flourishing Scale (Diener et al., 2010)

The 8-Item Flourishing Scale was chosen to examine well-being due to its positively worded items. It provides a single psychological well-being score by assessing positive and negative life experiences, as well as perceived successful relationships, self-esteem, purpose, and optimism (Diener et al., 2010). It also has good validity and correlation with overall well-being. For example, Diener et al. (2010) tested the validity of the scale using the Satisfaction with Life Scale (Diener et al., 1985) and Fordyce’s (1988) single-item measure of happiness, with positive correlations associated with overall well-being. The scale consists of 8 statements, including, ‘I lead a purposeful and meaningful life’ (purpose), and ‘I am optimistic about my future’ (optimism). Participants rate their agreement to the statements (1 = ‘strongly disagree’ to 7 = ‘strongly agree’). Total score ranges = 8–56. Higher scores indicate ‘flourishers’ defined as those who experience high levels of personal growth and feelings of happiness (Vittersø, 2004).

### Interview Schedule

The interview schedule (Supplementary Material 2) was developed by consulting previous literature (e.g., Conroy and de Visser, 2014; Jacobs et al., 2018) and examining research questions. Introductory questions were included at the start of the interview to build rapport (e.g., ‘Would you be able to tell me the main reason for being a person who drinks little or no alcohol?’). It was checked how participants identified (e.g., teetotal, sober, minimal drinker, the person who does not drink, etc.). The interview was split into three parts: arriving and settling into University, being around other drinkers, and friendship and social life. It was aimed that interviews would not take longer than an hour. Some questions were altered depending on people’s varied experiences (e.g., the person who had never drunk alcohol could not reflect on any change in drinking practices). Prompts were available to encourage more detailed responses, allowing the participant to pursue narratives that were of interest to the interviewer (Braun and Clarke, 2013).

### Piloting

Before use with participants, the interview schedule was piloted with three third-year students (two female medical students and one male history student). Piloting helped finalise question wording and flow. See Supplementary Material 3 for a summary of amendments after piloting.

### Data Collection and Procedure

Interview and survey data were collected by researcher EC (female, third-year medical student, University of Nottingham). Due to her medical training, EC was aware of principles of communication including rapport building, non-verbal communication, and question structure. Relationships with participants were not formally established before the interview aside from e-mail contact to book the interview slot. Participants were met either on Microsoft Teams (video-call) or were called on their mobile phone (phone number was taken beforehand *via* e-mail and deleted immediately after the interview). No one else was present on the call except EC. Demographic information, such as gender, age, and nationality, was collected before the interview *via* the third online survey, alongside the completion of the SPS-24 and Flourishing Scale. At the start of the interview, participants were reminded of the project aims and asked if they had understood the Participant Information Sheet (containing information about project title, the purpose of the study, what the study involved for them and any risks or benefits, and details about how their data would be handled) and Consent Form. Participants were made aware that they could disclose as much or as little as they like that there are no ‘right’ or ‘wrong’ answers and they can pause the interview if they felt uncomfortable or withdraw from the project up until December 18, 2020. They were reminded that the conversation was kept confidential within the research team and were asked if they had any questions before starting. Information provided before the interview included details of the interview format (i.e., questions surrounding the topic of not drinking), including their reasons for not drinking, how this has affected their transition into university, and whether it has affected friendships or relationships with other students. All participants were asked if they would like to know EC’s experience with alcohol before the interview schedule commenced. An audio recording (no video) of the interview was made by a Dictaphone and transcribed verbatim by EC. Transcriptions were not sent to participants for comment or correction. Field notes were kept for reference during coding. Participants were thanked for their time and were given the chance to ask any follow-up questions. They were all e-mailed a debriefing sheet which signposted relevant support services if needed. Interviews were deleted from the Dictaphone once transcribed.

### Data Analysis

During transcription, all participants were given a pseudonym and identifying features were removed (names and places). Although noted as a difficult concept to define (see Fusch and Ness, 2015), data saturation was deemed to be reached when no new information was gathered from the interviews (Guest et al., 2006).

An effort was made to use open questions and avoid leading questions if possible. We aimed to capture abstract ideas (e.g., personal perceptions) rather than concrete positive and negative terms that would not have allowed for flexibility (Hennink et al., 2017). Braun and Clarke’s (2006) six-step method of Thematic Analysis (TA) was used to analyse the transcripts. It is considered an effective method that enables the identification of reoccurring patterns within data (Braun and Clarke, 2006). An inductive approach was taken as it derives themes from the interpretation of participants’ experiences (Braun and Clarke, 2013).

Microsoft Word software was used for analysis. Transcripts were familiarised by EC by listening to the audio recording of the interview during transcription then reading the typed transcripts several times. Interesting features of data relating to the research project were identified, interpreted, and noted in the margin of the Word document. Features were organised into clusters with similar trends and summarised into codes (labels that are of importance to the research question) to create theme names. Connections were made between themes, with the allowance for the generation or collapse of others. Once reviewed to avoid missing important details, nine final themes were defined and collated to generate a codebook. The codebook aimed to summarise the themes identified by creating shared meanings of the data. Advantages of using Microsoft Word were, the computerised format was easy to manage and organise quotations as opposed to physical paper copies due to the large quantity. Microsoft Word allowed for notes to be added to the margins when highlighting individual quotations. This allowed for ease of access to quotes and themes. The ‘search’ feature helped find quotes.

Illustrative quotes were selected to provide participant voices to themes and sub-themes (see Eldh et al., 2020 for a detailed discussion about quote selection). These were predominately selected by EC, with verbal and written feedback from the supervisory team (KAJ and EBD).

Analysis was predominately conducted by EC, with supervisors (KAJ and EBD) providing a sounding board to discuss ideas and potential themes *via* regular weekly virtual meetings. As well as verbal feedback, both supervisors provided written feedback on EC’s proposed thematic analysis. However, there is still a risk of limited interpretation and assumption of objectivity (Silverman, 1993). To overcome this, 20 randomised quotations were given to two colleagues (TO and JC) to match the relevant themes and subthemes identified in the codebook with no input from EC or other researchers. Inter-rater reliability of 0.88 was calculated (one colleague scored 18/20 and the other scored 19/20; scores were added and converted into a decimal).

After analysis, due to time constraints, participants did not give feedback on the transcripts, codebook, or themes.

Raw scores of the SPS-24 and Flourishing Scale were linked to each participants’ pseudonym to provide a descriptive account of the student’s well-being (flourishing) and perceived social provisions.

### Research Team and Reflexivity

In their update on thematic analysis, Braun and Clarke (2019) stressed the importance of reflexivity. The background, views, and beliefs of the research team about the topic of drinking little or no alcohol will be described here. Researcher EC describes herself as someone who drinks little alcohol. EC came to University as a non-drinker and has first-hand experience of navigating the initial transition period of not drinking alcohol. EC specifically experienced social barriers during Fresher’s week and in sporting societies. She has friends who do not drink for other reasons and has seen how that has affected them. EC has a specific interest in exploring the stories of other students who drink little or no alcohol within a University drinking culture.

Supervisor KAJ (female) is a former drinker who now drinks no alcohol. She navigated University as a drinker. She has both an academic and personal interest in the topic and is interested in the positive experiences of people who do not drink alcohol.

Supervisor EBD (female) is a current minimal drinker, who entered University as a non-drinker and started drinking during university. EBD has an academic and personal interest in this topic, specifically in how people who drink little or no alcohol navigate their decisions.

## Results

After screening (Survey 1), 20 participants were eligible for the study. Fifteen participants completed Survey 3 and were interviewed. The remaining five did not consent and complete the study (no reasons given).

The final sample included 15 students who were interviewed (12 women, three men, mean age = 21, *SD* = 0.97, range = 20–23). The majority were based in the Faculty of Medicine and Health Sciences (*n* = 9), with *n* = 3 based in the Faculty of Science, *n* = 2 in Engineering, and *n* = 1 did not describe. Interview length ranged between 18 and 38 min (*M* = 30.73, *SD* = 6.16). All participants agreed to hear the interviewer (EC)’s experiences with alcohol. No repeat interviews were conducted. Supplementary Figure 1 outlines the flow of participation.

Table 1 shows demographic information about the participants. Students were mostly in year three of their studies (year three *n* = 8, year four *n* = 4, year five *n* = 2). The overall mean total SPS-24 score was 81.67 (*SD* = 11.42, range = 48–93). Four participants scored 90 or above, six scored above 80, and three scored above 70 with two remaining participants scoring 48 and 69. Detailed subscale scores for SPS-24 for each student are provided in Supplementary Table 1 and will be used, if relevant, alongside their quotes. The overall mean FS score was 47.80 (*SD* = 6.89, range = 34–56), indicating generally high levels of flourishing among participants.

**TABLE 1:** Participant demographics including total SPS-24 and FS scores.

Pseudonym	Year	Ethnicity	Self-described drinker ‘status’	Method of interview	Total SPS-24 score^1^	Total FS score^2^
Harry	4	White British	Former drinker	MS teams	48	34
Mia	3	Asian/Vietnamese British	Someone who drinks little alcohol*	MS teams	92	49
Amir	3	Asian	Someone who has never drank alcohol	MS teams	84	44
Zoe	4	White Irish	Someone who drinks little alcohol*	MS teams	85	40
Aisha	3	Pakistani	Someone who has never drank alcohol	Phone	87	55
Sophie	3	White British	Someone who has never drank alcohol	MS teams	73	38
Iman	4	Indian	Someone who drinks little alcohol*	MS teams	93	55
Emma	5	White British	Someone who drinks little alcohol*	MS teams	93	54
Olivia	3	White British	Someone who drinks little alcohol*	MS teams	79	47
Danielle	3	Black British	Someone who drinks little alcohol*	MS teams	76	46
Amara	3	British Indian	Someone who drinks little alcohol*	MS teams	88	56
Lily	5	White British	Former drinker	MS teams	69	50
Grace	4	Mixed White/Black African	Someone who has never drunk alcohol	Phone	91	52
Kate	4	White	Someone who drinks little alcohol*	MS teams	82	41
Jess	3	Arab	Someone who drinks little alcohol*	MS teams	85	56

Higher SPS-24 scores indicate elevated levels of perceived social support. Higher FS scores indicate elevated levels of social well-being, perceived successful relationships, and optimism. *Scored three or below on the AUDIT-C.

*^1^Total SPS-24 score is 96, using reverse-scoring.*

*^2^Total FS score is 56.*

### ‘Type’ of Non-drinker and Reasons for Not Drinking Alcohol

The majority of participants drank little alcohol (*n* = 9, all scored three or below on AUDIT-C), followed by people who had never consumed alcohol (*n* = 4) and former drinkers (*n* = 2). Former drinkers scored a mean of 58.5 (*SD* = 14.85) on the SPS-24 and 42 (*SD* = 11.31) on the FS. People who drank little alcohol scored a mean of 85.89 (*SD* = 6.17) on the SPS-24 and 49.33 (*SD* = 6.28) on the FS. People who had never consumed alcohol scores a mean of 83.75 (*SD* = 7.72) on the SPS-24 and 47.25 (*SD* = 7.72) on the FS.

The interviewer learnt more about participants’ reasons for their decision to drink little or no alcohol through the interview. Most participants (*n* = 10) felt there was no need to drink alcohol. Reasons included disliking the taste (*n* = 3), the price of alcohol (*n* = 4), health-related reasons (*n* = 2), and lack of interest. Most felt it would not improve their enjoyment. Some participants described not having many external pressures from family and friends before University. For example, growing up with peers who are not motivated to drink alcohol do not have an expectation to drink. Others (*n* = 11) were influenced by religious and cultural beliefs. Nine of these participants explained how drinking would cause problems within their faith, the remaining two were never exposed to alcohol at home.

Over half (*n* = *9*) participants in the study described the main reason they did not drink alcohol was that they felt unsafe or out of control. Some described other moments of their life where they felt out of control, affirming their disinterest with alcohol. Participants described not wanting to experience the consequences of alcohol. For example, they did not want to burden their friends, expressing feelings of embarrassment. Finally, three female participants (*n* = 3) described feelings of vulnerability in drinking environments. One participant revealed she previously had her drink spiked. Consequently, these participants described acting with caution to try to remain aware of their surroundings. Finally, one participant briefly mentioned his dad holding similar qualities to an “alcoholic,” although he did not explicitly describe his dad as a “typical alcoholic,” but someone who drinks more than usual – this was not discussed further as it was not relevant to the participant’s experience at University, However, it did influence his decision not to drink as he did not like how his dad acted when drunk.

### Summary of Themes and Subthemes

Eight main themes and four subthemes were generated from thematic analysis (see Table 2). Quotes from participants are presented with their total SPS-24 and FS scores to give further context about social connectedness and flourishing. To emphasise novel findings, these themes have been organised into two sections; ‘views of drinkers from non-drinkers’ and ‘how peer pressure feels and how people deal with it.’

**TABLE 2:** Summary of eight main themes and four subthemes included in two overarching sections.

Section	Main theme	Subtheme
Views of drinkers from non-drinkers	1. Sobriety filters friendships.	
		6a. The fear of being ‘boring’
	7. Drunk people are on another level.	7a. Going past tipping point
	8. Perceived effects of alcohol and life as a non-drinker	
How peer pressure feels and how people deal with it	2. An uncomfortable situation	2a. They just don’t get it
	3. Loosening the grip of peer pressure	
	4. The ‘typical’ student lifestyle	
	5. A gradually improving experience	
	6. The battle between self-assurance and self-doubt	6b. What if…?

#### Views of Drinkers From Non-drinkers (Includes Themes 1, 7, and 8 and Subtheme 6A)

Themes in this section generally describe how drinkers are viewed by non-drinkers. For example, *Theme 1. Sobriety filters friendships* most participants (*n* = 11) explained how their closest friends share similar interests in that they are also not overly interested in drinking and do not rely on alcohol to enjoy themselves:

*‘the people in my circle aren’t the type of people that enjoy going out and getting drunk*… *a lot of my friends would rather go out for lunch or coffee and be in bed in the evening’* – Sophie (SPS-24 = 73, FS = 38).

Several participants (*n* = 6) explained the process of making friends took longer as they had to sort their ‘true’ friends from those who could not understand their choices.

*‘I probably would have been closer friends with certain people.*…*I know some people just don’t rate that I don’t get drunk, they just don’t*… *understand it because they*… *might think it’s a bit boring’* – Iman (SPS-24 = 93, FS = 55).

In *Subtheme 6A. The fear of being ‘boring’* participants (*n* = 9) revealed worries of feeling ‘boring’ or didn’t feel confident to reveal their drinking status in every situation, especially if around drinkers. For Harry, this was discussed about meeting a romantic partner:

*‘I do sometimes build it up*… *at some point they’re going to find out*…*There’s someone who I’m speaking to at the moment who I’ve not said [I don’t drink] yet, and I would probably try to hide it from him for quite a while’* – Harry (SPS-24 = 48, FS = 34). Harry also had relatively low scores on some SPS-24 subscales (e.g., attachment, nurturance, reliable alliance, and guidance) in comparison to his peers (see Supplementary Table 1).

Participants (*n* = 4) described how peers (who drank alcohol) resorted to stereotypes before they got to know them. For example, in this case, Sophie felt people viewed her as judgemental due to her Christian beliefs.

*‘as a Christian, if I told them that I didn’t drink, then they’d think I was some judging monster. Like, I am perfect, I am holy*,…*and I am condemning you. I was really worried to come across like that’* – Sophie (SPS-24 = 73, FS = 38). It is also interesting to note that Sophie also had a lower score on attachment (score = 5) on the SPS-24 in comparison to most of the other students in the study.

In *Theme 7. Drunk people are on another level* participants (*n* = 10) described in more detail why they did not enjoy going to clubs. It was felt there was a ‘language barrier’ between drinkers and non-drinkers, finding it hard to socialise with someone who was *“not on the same level as them”* (Jess). Participants often felt they could not stay as late as their peers and preferred pub environments where conversations could still be held:

*‘I really quickly didn’t like it because you didn’t really get to talk to people because it wasn’t really like going to a pub or something*… *how good a conversation can you have with someone in a club?’ – Olivia* (*SPS-24* = *79, FS* = *47*).

Experiencing a struggle to connect to drunken peers (*n* = 7), participants would distance themselves. However, over half (*n* = 10) expressed having another friend who was not drinking made this easier:

*‘it was nice that we both weren’t [drunk] cause then we just had each other, and it was fine, whereas everyone else was drinking and was like, gone’ – Lily* (*SPS-24* = *69, FS* = *50*). Lily also had a relatively low score (score = 8) in comparison to most of her peers on subscale nurturance in the SPS-24, which describes people’s feelings of others depending on them for help.

Danielle, a former drinker, described feeling able to connect with other drinkers as she can share her own experiences and therefore feels included in conversations:

*‘I can look back, like when we’re talking about drinking*…*and tell stories from when I was in school. It’s not like I’m completely isolating and have never ever had an experience of drinking alcohol’ – Danielle* (*SPS-24* = *76, FS* = *46*).

In a smaller theme (*Subtheme 7A. Going past tipping point*) participants described drinkers reaching a certain level of drunkenness, and subsequently losing control. Frequently (*n* = 7), participants spoke negatively about their peers’ behaviour, expressing irritation that they “can’t handle their drink” (Zoe), ruining their fun and assigning immediate responsibility on them:

*‘if you’re the only sober person, you end up having to take care of people. Which is fine [occasionally], but sometimes*… *I have to end the night out there at 12 pm*… *it’s a bit annoying’ – Amara* (*SPS-24* = *88, FS* = *56*).

This disinhibited behaviour affirmed participants’ choices not to drink:

*‘working with individuals at nights out [and] with the ambulance service*… *you see a lot of drunk students*…*well, just drunk people, and I’m just like ‘I really don’t want to ever be in that state’ – Amir* (*SPS-24* = *84, FS* = *44*).

*Theme 8. Perceived effects of alcohol and life as a non-drinker* provides a summary of descriptions of drinkers which were generally negative. Drinkers were viewed as immature, relying on alcohol to boost their confidence. Some participants (*n* = 5) felt they were ranked by others on *‘how fun you are based on how often you go out’* (Kate), as others used terms such as *‘heroes’* to describe drinkers. Participants described the sense of social status elevation by drinking:

*‘I find that the majority of people that I meet will drink*… *for the sake of drinking and getting drunk and wanting to be unwell the next day, cause you know, there’s a badge of coolness or whatever’ – Kate* (*SPS-24* = *82, FS* = *41*).

Two former drinkers described they were treated no differently when they drank, yet others (*n* = 2) felt others portrayed them in a ‘cooler’ manner:

*‘when I did drink*,…*some people would act differently towards me*… *[they] felt more comfortable to come up to me because they’d know I’d be on the same level*…*they saw me in a cooler way’ – Jess* (*SPS-24* = *85, FS* = *56*).

Other analogies were used to describe their frustration with the drinking culture, where there should be no ‘norm’:

*‘I feel like people can’t really have an issue anymore*…*you wouldn’t force someone who’s a vegan to eat a sausage*… *so why would you force someone to have a drink?’ – Sophie* (*SPS-24* = *73, FS* = *38*).

#### How Peer Pressure Feels and How People Deal With It (Includes Themes 2, 3, 4, 5, 6, and Subtheme 6B)

The themes in this section describe the experience of peer pressure, how it changed over time, and how students coped. In *Theme 2. An uncomfortable situation* two participants described feeling unphased by peer pressure, but all participants could give at least one example of being pressured to drink by their peers:

*‘It was such an uncomfortable situation*… *I guess that was the one time that, actually to fit in, you almost, not had to*… *but it was very much ‘you’re going to be drinking”* – Harry (SPS = 48, FS = 34).

Participants tended to put peer pressure along a gradient. Two described it to be overt and ‘forceful’ in nature, others (*n* = 4) felt it was more ‘gentle’ encouragement. Here Iman describes how their peers may not understand the more subtle pressure they inflict.

*‘they don’t actually wish harm*… *they’re not being serious, but if you don’t know them, it can come across like they’re trying to be a bit pushy’* – Iman (SPS-24 = 93, FS = 55).

Several (*n* = 3) expressed curiosity from friends about the “type of drunk” they would be. Here Aisha, a person who has never drunk alcohol describes her experience:

*‘everyone just wants to see what type of drunk you are and if you haven’t drank so they can try and convince you’* – Aisha (SPS-24 = 87, FS = 55).

When the pressure was indirect, participants described feeling guilty if they did not drink. Kate, who drinks little alcohol, describes the feeling of having to fit in to make friends:

*‘I did meet people in my first year*…*trying to put me in these situations as sort of ‘the bargain for friendship is that you have to come out and drink with us” –* Kate (SPS-24 = 82, FS = 41).

In *Subtheme 2a. They just don’t get it* participants expressed frustration about the assumption that everyone drinks at University. Many (*n* = 11) experienced further questioning from their peers when people noticed they did not drink. Here, Olivia describes it as accusatory.

*‘I guess sometimes as well people pose a question in a way as if like, they find it weird that you’re not drinking*… *they’d be like, ‘why,’ more accusatory I guess, like, ‘why aren’t you drinking?’* – Olivia (SPS-24 = 79, FS = 47).

If they did not have a specific reason for not drinking, participants felt it was more difficult to respond to questions from peers. The shared experience of drinking appears to be a ‘common ground’ and a way to start a conversation with a fellow student. Here, Danielle (who now drinks little alcohol) gives an example of a conversation they felt was shut down due to a lack of shared interests.

*‘they’d ask…, ‘oh, where have you been out?’*… *cause that was just kind of like an opening line. When you’re like, ‘oh, I don’t really go out a lot,’ you feel like they don’t really know what else to say to you’* – Danielle (SPS-24 = 76, FS = 46).

Nine participants described experiencing no pressure to drink from closest friends, of which seven would avoid conversations of alcohol when with acquaintances, or try to find alternative reasons for not drinking through a lack of understanding:

*‘I think most people, to be honest, are quite accepting of it and can understand that. But then there would be a couple of people who would be like, on no, there’s bound to be something else, you know’* – Zoe (SPS-24 = 85, FS = 40).

*Theme 3. Loosening the grip of peer pressure* describes finding ways to overcome external pressure. All participants spoke about making excuses or having a ‘valid’ reason for not conforming to the ‘typical’ student lifestyle:

*‘I did find myself making lots of excuses for why I wasn’t going out.*…*I was saying I was feeling a bit sick*…*when realistically, I just didn’t wanna go out’* – Zoe (SPS-24 = 85, FS = 40).

Not drinking for religious reasons was discussed by several participants. Sophie described how she felt she would be judged as a Christian non-drinker. Aisha, one of three Muslim students participating in the study, felt her religion was not a valid excuse anymore.

*‘I feel like [being a Muslim] used to be more of a valid reason*… *before if I would just say I’m a Muslim, that was enough, but now I feel like it’s just not’* – Aisha (SPS-24 = 87, FS = 55).

Seven participants coped by lying about their drinking or deferring questions to reduce the possibility of pressure. Others (*n* = 5) would make or order drinks that visually looked alcoholic. In this example, Emma asks for a drink to be made with a small amount of alcohol.

*‘I went up to the bar and asked them if they could make me a vodka and orange with like, a quarter of a shot of vodka in it*… *one person asked*…*and I said ‘oh, vodka and orange,’ without mentioning the amount of vodka’* – Emma (SPS-24 = 93, FS = 54).

Some participants described that by showing confidence in their decision, they were respected for their choice. However, if they had consumed alcohol before, their peers saw it as an easier barrier to break down and convince them to drink:

*‘if you just said you were teetotal…, no one would pressure you*… *but if you show*… *flexibility, they’re more likely to try because they know that they can get somewhere with that’* – Iman (SPS-24 = 93, FS = 55).

Two participants described pre-empting perceived opinions by joking about their behaviour. By acknowledging their outsider position as a non-drinker in a drinking environment, they tried to divert potential stigma and control the narrative. Sophie, a person who has never drunk alcohol gives an example.

*‘I would have Pepsi Max or squash*…*and I made a point of it being funny and it being a joke.…instead of letting them say anything, it was just a joke anyway’* – Sophie (SPS-24 = 73, FS = 38).

In *Theme 4. The ‘typical’ student lifestyle* self-imposed expectations to drink at University were commonly described by participants (*n* = 8). Despite many participants displaying confidence in their decision not to drink (*n* = 10), not conforming to the ‘typical’ student experience led to feelings of being different or *‘doing uni wrong’* (Kate). This was revealed in every interview. For example:

*‘it’s always a bit weird to be like, someone that doesn’t drink. Well, not weird, but you know, different, isn’t it?’* – Grace (SPS-24 = 91, FS = 52).

Participants expressed frustration about the University stereotype, giving examples of negative portrayals of students in the media. As a result, three participants felt the drinking culture was wildly exaggerated:

*‘I wish there would be less things in the news about students who are drunk*… *alcohol and being a student are synonymous of each other and people don’t imagine there are students that don’t drink*… *it shows us as someone who’s out of control and irresponsible*… *which is obviously not the truth’* – Aisha (SPS-24 = 87, FS = 55).

However, in *Theme 5. A gradually improving experience* participants felt their experience of settling in and the acceptance from peers greatly improved over time. Their peers required less approval from them, which participants attributed to maturity (see theme 8), where the novelty of alcohol began to wear off:

*‘as time went on, it was funny seeing the change because they were never bothered me not really doing it because like, you know, I would do other things*… *the novelty wore off for them’* – Sophie (SPS-24 = 73, FS = 38).

Kate explained with dissatisfaction how, only now, does it “feel how it should” for non-drinkers, suggesting stereotypes are slowly lifting. There was a previous embarrassment for being a non-drinker:

*‘but back then in first year, it genuinely seems like the worst think in the world for people to know that you don’t like to drink’* – Zoe (SPS-24 = 85, FS = 40).

Since this study was carried out during a national lockdown, participants felt this had improved their experience of University further:

*‘I think lockdown massively helps, in the sense that we’re not going out so*… *I don’t feel like I need to drink or anything like that’* – Jess (SPS-24 = 85, FS = 56).

Students talked about their personalities and characteristics and how this interacted with their drinking status. In *Theme 6. The battle between self-assurance and self-doubt* a sense of self-assurance was revealed in every interview. Mia describes feeling more certain of her decision after exploring the University drinking culture.

*‘I feel like now that I know what [clubbing’s] like, I feel more kind of affirmed in my type of lifestyle. I feel like I was quite content and happy without having to rely on alcohol too much, like in a social setting’* – Mia (SPS-24 = 92, FS = 49).

By displaying a confident and chatty personality, most (*n* = 8) participants described how they do not need to rely on alcohol in a social setting like others:

*‘I can hold a conversation without alcohol, and I think that’s a big confidence thing as well, like some people need alcohol for a confidence boost’* – Danielle (SPS-24 = 76, FS = 46).

Three participants described feeling as though they do not need to justify themselves anymore. In this case, Aisha attributes it to the COVID-19 pandemic when going out is not an option.

*‘Since the pandemic, I’ve noticed I’ve stopped justifying a lot, like before I would have to give a*… *reason to explain myself*… *I don’t need to justify it anymore, it’s just so exhausting coming up with a list of reasons*’ – Aisha (SPS-24 = 87, FS = 55).

Additionally, the *Subtheme 6B. What if…?* related to a curiosity to drink and a sense of ‘what if.’ For example, many (*n* = 10) felt they would have made alternative friendships if they drank alcohol. Five participants were interested in experiencing alcohol’s effects, two of which were minimal drinkers. They did not want to feel the consequences, such as a lack of control, but there was a sense of *‘wanting to know what all the hype was about’* (Kate):

*‘I don’t know what the fascination is. I’m a bit curious to see*,…*I want to know how it feels, but I don’t want the problems’* – Amara (SPS-24 = 88, FS = 56).

Some participants (*n* = 3) felt they did not have the ‘liquid courage’ to be more sociable and were therefore too aware to ‘loosen up.’ This created difficulties when meeting potential romantic partners:

*‘when you’re sober and you’re in a nightclub, I feel like my guard is up a lot more than obviously my friends’ were. So maybe if I had been looser in certain situations, things could have happened’* – Grace (SPS-24 = 91, FS = 52).

## Discussion

This study aimed to explore the role of sobriety in the transition to and through University and provide an overview of general levels of flourishing and social provision in this self-selected group. Overall students had high levels of flourishing and social provision, this was supported by quantitative accounts with students describing their enjoyment of close friendships and fewer casual acquaintances (Theme 1: ‘sobriety filters friendships’). Students talked about their views of drinkers from the perspective of a non-drinker, they described being on a different level to people when they were drinking, making the connection with other people more difficult, and held negative perceptions of some drinkers. Students encountered both overt and covert peer pressure to drink alcohol and, as reported in other studies, developed coping mechanisms to deal with such pressure. Some students reflected how their drinking status meant they had grown in confidence over time. Representing a diverse case (Harry), drinking little or no alcohol was described as more difficult to navigate when looking for a romantic partner. Harry also had relatively low scores on subscales reliable alliance, attachment, guidance, and nurturance on the SPS-24 (see Supplementary Table 1) in comparison with other participants in the study, potentially indicating overall feelings of lack of social connection.

Overall, themes mirrored previous UK-based research, where students described being viewed by drinkers as a homogenous group or stereotype. Stereotypes of people who drank little or no alcohol intersected with other characteristics such as religious beliefs (Piacentini and Banister, 2009; Banister et al., 2019). For example, ‘the judgemental Christian.’ Such quick judgement from peers possibly represents automatic stereotyping processes (organising others into categories without forethought) due to meeting so many new people at once at University. As explored in other qualitative studies (Conroy and de Visser, 2014, 2018), stereotyping raises concerns of alienation and exclusion. The ‘unsociable’ stigma associated with non-drinking (Supski and Lindsay, 2017; Conroy and de Visser, 2018) was present in the narrative of students. Findings mirrored Herring et al.’s (2014), as students described how they felt morally obliged to take on a caring role if others were intoxicated, overshadowing their enjoyment. Overlaps were identified between the expectations to drink at University, in this study, and Nairn et al.’s (2006) research, where non-drinkers felt they needed to justify their decision or conceal their identity because they were not adhering to the ‘typical’ student lifestyle. The theme ‘loosening the grip of peer pressure’ described similar attempts made to minimise these stigmatic associations (Conroy and de Visser, 2014; Zhou and Heim, 2016; Jacobs et al., 2018). Overall, findings typically agreed with previous research, but this study allowed further expansion on building relationships, pressure, and overcoming counter-normative behaviour.

The experience of internal and external pressure to drink in this study was complex. All participants gave at least one example of peer pressure, though rarely felt it was ‘extreme’ (Harry) or forceful and was never from their closest friends. Instead, participants described gentle encouragement, mainly from casual acquaintances who attempted to break down participants’ ‘problems’ (Conroy and de Visser, 2014). One participant (Danielle) felt that having no particular reason resulted in further problematisation as the required explanation could not be given. Deception was described by some to cope at that moment, for example, ordering drinks that looked or smelt alcoholic (Emma) or pretending to feel unwell. Aisha described her Muslim identity no longer felt ‘valid’ as a reason for not drinking. This contradicts Zhou and Heim’s (2016) idea of a ‘fractured in-group’, where religion should constitute a valid reason. These results may be due to the modernisation of faith and an increase in the proportion of people of the Muslim faith who drink (Hurcombe et al., 2010). Participants reported curiosity from others about the ‘type of drunk’ they would be (internally experienced from those who had never drunk alcohol), supporting an underlying expectation to drink (Nairn et al., 2006; Piacentini and Banister, 2009). All participants felt the most pressure came from themselves, for not conforming to the norm. Participants often blamed the media for exaggerating the University, resulting in feelings of “doing uni wrong” (Kate), particularly during Fresher’s week. We know students feel pressure to get drunk at University, but studies have not explored in detail how media reporting of student behaviour may contribute to these expectations.

Some themes identified in this study were unexpected, reflecting novel information. Additional attempts to minimise peer pressure were exhibited by two overlapping traits. Some participants described feeling emotionally equipped, being able to take control of possible pressures, and therefore adopting a ‘self-assured’ trait. While others had the tendency to ‘self-doubt,’ attempting to avoid conversations about alcohol when in potentially ‘dangerous’ (e.g., potential social exclusion) situations. For ‘self-assured’ participants, by displaying outward confidence in social situations, their decision not to drink was respected. These findings connect with Conroy and de Visser’s (2014) idea of ‘weak = easy,’ where less pressure is applied to non-drinkers if they stand firm. One participant (Iman) believed showing flexibility toward alcohol increased the likelihood of pressure, as there was more scope for breaking down the barrier. This is reflected in Patrick and Hagtvedt’s (2012) theory of ‘goal-directed behaviour,’ where using ‘don’t’ rather than ‘can’t’ drink is more psychologically empowering. Secondly, by acknowledging potential stigmatisation, Sophie described pre-empting pressure by mocking her behaviour before others could. There is no doubt that humour has a social function (e.g., Martineau, 1972). However, self-deprecating humour may have an impact on a person’s self-esteem and well-being over time.

### Strengths and Limitations

The researcher offered to share their own experience with alcohol (previous non-drinker), which all participants agreed to hear. Such a technique is aligned with ‘peer interviewing’ where the interviewer (in this case an undergraduate student with experience of navigating university as a person who drinks little or no alcohol) is part of the same ‘group’ as the interviewee (Devotta et al., 2016). Such an approach has pros and cons. It may have facilitated a more comfortable environment for participants but altered participants’ narrative as they may have shared views or experiences, they deemed to be ‘correct’ in an attempt to mimic the interviewer’s experience. Despite awareness of potential confirmation bias, the interviewer’s interpretation of results is perhaps more favourable toward their own previously held beliefs. Although the interviewer can empathise with participants, their similarity in age and stage at University may have allowed for a deeper connection and more rich discussions. Both supervisors had the experience of not drinking alcohol or identified as ‘teetotal’ or a ‘minimal drinker’ at various stages of their lives which is a strength as they can relate to some of the experiences of students but as they are comfortable in their choices, may have biassed the analysis to see positive aspects of not drinking.

It is noted this study has a small sample size with only three men taking part in the study. As found in previous research, the pressures on men may be different for women (e.g., De Visser et al., 2009). There was also no representation of people identifying as *trans* or non-binary, which is a gap in the literature. Students in the study represented undergraduate students who were further along in their university experience (years three to five), which was a strength as it allowed them the opportunity for reflection (a novel aspect of our study). However, the voices of year two (who may have a better memory of the Fresher experience) were not heard. This may have been due to the researcher being in year three of her studies, the way the study was advertised, or potentially year two students not being as confident to come forward and discuss their experience as those in later years of their study. Future studies should focus on recruiting second-year students into such studies to ensure their valuable insight is heard. Though those in later years were able to reflect on the initial challenges of the transition into University including finding it longer to build strong relationships with peers and find their ‘true’ friends. Having found such friends, they were able to confirm or deny true friends by people’s reaction to their choices, a position other who had few close friends may not have been able to take. Time constraints to collect the data also introduced some limitations. For example, only students in UoN were recruited for this study, therefore the experiences of these students may not be generalisable to students attending other UK universities. Further, due to timing constraints transcripts and themes were not checked by participants. Over half of the participants were based in the Faculty of Medicine: it is possible that due to their degree of study, these students may have been more aware and understanding of the effects of alcohol, and more inclined to drink little or no alcohol. Future research may wish to explore the experiences of students based in non-medicine faculties.

The majority of participants (60%) in this study were not completely sober or teetotal and drank small amounts of alcohol infrequently (maximum of 4 units less than monthly). It could be argued that these students could not provide adequate insight on sobriety by its formal definition (no consumption of alcohol). It could also be argued that these students may have changed their behaviour (decided to drink a small amount of alcohol rather than abstain) due to peer pressure or being immersed in the culture of University but may not have appropriate insight about this while still in this context. However, the views of people who have never consumed alcohol (*n* = 4), and former drinkers (*n* = 2) added alternative viewpoints to the analysis. Notably, those who had never drunk alcohol expressed curiosity toward its effects. Former drinkers had the lowest scores on the SPS-24 and FS compared to the other two groups, but the sample size was small. Despite the aim to capture a variety of experiences, the students in the sample who volunteered to take part may be more likely to hold their decision as part of their identity and feel comfortable discussing these decisions with a stranger. Those socially isolated, struggling with their identity and decisions (all of which may impact well-being) may be more reluctant to participate, reducing the richness of results. Future studies should focus specifically on engaging people who were either undecided about their decision or were more socially isolated.

Quantitative measures collected in the study aimed to contextualise the qualitative data by providing further detail about flourishing and social provisions. Overall, participants displayed high levels of well-being and felt they had a good range of social support, something which was not fully discussed in interviews. Although providing a relative score for each individual makes comparison easier than a subjective interpretation of experiences, these results are insufficient by themselves. A quantitative study with the opportunity for students to provide comments (content analysis) may be appropriate in future studies, as it may recruit a larger sample and allow for quantitative analysis to explore relationships between well-being and social provision. Given the time frame, transcripts were not reviewed by participants or read by other researchers, which could have allowed for more varied interpretations.

The most notable influence on this study was COVID-19, where national restrictions at the time prohibited in-person interviews. It is not known how the physical presence of a face-to-face interview may have influenced the results. The online setting may potentially have influenced the interview process: the *online disinhibition effect* refers to how people may be less restraining in online environments (Suler, 2004; Lapidot-Lefler and Barak, 2012), meaning participants were more likely to self-disclose and share personal experiences online than in the offline world. However, the absence of in-person body language may have limited the rapport built during the interview. The recruitment process (moved online due to COVID-19) was a probable cause for the high dropout rate (31.8%) due to their length of completion and inconvenience. If the study were repeated without COVID-19 restrictions, the FS and SPS-24 questionnaires could be conducted at interviews. Data for this study was collected during national social restrictions due to COVID-19, which limited the breadth of advertising and snowballing – as well as having a general impact on students’ mental health.

### Conclusion

Though students interviewed were generally flourishing and socially connected, they still described overt and covert peer pressure to drink, adopted strategies to fit in, and felt they were stereotyped and questioned about their choices by drinkers. University drinking culture and media portrayals of student behaviour were seen as fuelling internal and external pressure to drink or risk not having the ‘true’ student experience. Future work should examine how student life is portrayed in the news media and social media, to establish how students who drink little, or no alcohol are represented. Moving away from ‘us’ and ‘them’ about drinking status would be an important goal to promote better understanding between peers when new at University which may reduce stereotyping. More work is needed by Universities to create inclusive spaces and activities, particularly during the early years when first establishing friendships.

## Data Availability Statement

The raw data supporting the conclusions of this article will be made available by the authors, without undue reservation.

## Ethics Statement

The studies involving human participants were reviewed and approved by the University of Nottingham’s Division of Psychiatry and Applied Psychology Research Ethics Committee (reference number: 1663). The patients/participants provided their written informed consent to participate in this study.

## Author Contributions

EC contributed to the design of the study and data collection and analysis and wrote the manuscript. ED contributed to the conception and design of the study, advised on data collection and analysis, and wrote the manuscript. KJ originally conceptualised the study, contributed to the design, advised on data collection, and wrote the manuscript. All authors contributed to the article and approved the submitted version.

## Author Disclaimer

The views expressed are those of the author(s) and not necessarily those of the NHS, the NIHR, or the Department of Health and Social Care.

## Conflict of Interest

The authors declare that the research was conducted in the absence of any commercial or financial relationships that could be construed as a potential conflict of interest.

## Publisher’s Note

All claims expressed in this article are solely those of the authors and do not necessarily represent those of their affiliated organizations, or those of the publisher, the editors and the reviewers. Any product that may be evaluated in this article, or claim that may be made by its manufacturer, is not guaranteed or endorsed by the publisher.

## Footnotes

1 National Union of Students Press Team. New survey shows trends in student drinking [Internet]. 2018 [cited 2021 April 1st]. Available from: https://www.nus.org.uk/en/news/press-releases/new-survey-shows-trends-in-student-drinking/

2 How much do people binge drink in Great Britain? – Office for National Statistics [Internet]. [cited 2021 April 1st]. Available from: https://www.ons.gov.uk/peoplepopulationandcommunity/healthandsocialcare/drugusealcoholandsmoking/articles/howmuchdopeoplebingedrinkingreatbritain/2015-06-12
