# Supplementary material for: “Drunk People Are on a Different Level”: A Qualitative Study of Reflections From Students About Transitioning and Adapting to United Kingdom University as a Person Who Drinks Little or No Alcohol
Source: Front Psychol. 2022 Jan 27;12:702662. doi: 10.3389/fpsyg.2021.702662 (PMC8829061; doi:10.3389/fpsyg.2021.702662)
Supplement: Supplementary file 1 [file Data_Sheet_1.docx]

**Supplementary material 1. Consolidated criteria for reporting qualitative studies (COREQ): 32-item checklist**

Developed from:

Tong A, Sainsbury P, Craig J. Consolidated criteria for reporting qualitative research (COREQ): a 32-item checklist for interviews and focus groups. *International Journal for Quality in Health Care*. 2007. Volume 19, Number 6: pp. 349 – 357

YOU MUST PROVIDE A RESPONSE FOR ALL ITEMS. ENTER N/A IF NOT APPLICABLE

| **No. Item** | **Guide questions/description** | **Reported on Page #** |
| --- | --- | --- |
| Domain 1: Research team and reﬂexivity |  |  |
| *Personal Characteristics* |  |  |
| **1. Inter viewer/facilitator** | Which author/s conducted the interview or focus group? | 6 |
| **2. Credentials** | What were the researcher’s credentials? E.g. PhD, MD | 6 |
| **3. Occupation** | What was their occupation at the time of the study? | 6 |
| **4. Gender** | Was the researcher male or female? | 6 |
| **5. Experience and training** | What experience or training did the researcher have? | 6 |
| ***Relationship with participants*** |  |  |
| **6. Relationship established** | Was a relationship established prior to study commencement? | 6 |
| **7. Participant knowledge of the interviewer** | What did the participants know about the researcher? e.g. personal goals, reasons for doing the research | 7, reflections on page 16 |
| **8. Interviewer characteristics** | What characteristics were reported about the inter viewer/facilitator? e.g. Bias, assumptions, reasons and interests in the research topic | Pages 7-8, reflections on page 16 |
| **Domain 2: study design** |  |  |
| ***Theoretical framework*** |  |  |
| **9. Methodological orientation and Theory** | What methodological orientation was stated to underpin the study? e.g. grounded theory, discourse analysis, ethnography, phenomenology, content analysis | 7 |
| ***Participant selection*** |  |  |
| **10. Sampling** | How were participants selected? e.g. purposive, convenience, consecutive, snowball | 5 |
| **11. Method of approach** | How were participants approached? e.g. face-to-face, telephone, mail, email | 5 |
| **12. Sample size** | How many participants were in the study? | 8 |
| **13. Non-participation** | How many people refused to participate or dropped out? Reasons? | 8 |
| ***Setting*** |  |  |
| **14. Setting of data collection** | Where was the data collected? e.g. home, clinic, workplace | 5 |
| **15. Presence of non-participants** | Was anyone else present besides the participants and researchers? | 6 |
| **16. Description of sample** | What are the important characteristics of the sample? e.g. demographic data, date | 8 and Table 1 |
| ***Data collection*** |  |  |
| **17. Interview guide** | Were questions, prompts, guides provided by the authors? Was it pilot tested? | 6 and Supplementary Material 2 & 3 |
| **18. Repeat interviews** | Were repeat inter views carried out? If yes, how many? | No, see page 8 |
| **19. Audio/visual recording** | Did the research use audio or visual recording to collect the data? | 7 |
| **20. Field notes** | Were ﬁeld notes made during and/or after the interview or focus group? | 7 |
| **21. Duration** | What was the duration of the interviews or focus group? | 8 |
| **22. Data saturation** | Was data saturation discussed? | 7 |
| **23. Transcripts returned** | Were transcripts returned to participants for comment and/or correction? | 7 |
| **Domain 3: analysis and ﬁndings** |  |  |
| ***Data analysis*** |  |  |
| **24. Number of data coders** | How many data coders coded the data? | 7 |
| **25. Description of the coding tree** | Did authors provide a description of the coding tree? | 7 |
| **26. Derivation of themes** | Were themes identiﬁed in advance or derived from the data? | 7 |
| **27. Software** | What software, if applicable, was used to manage the data? | 7 |
| **28. Participant checking** | Did participants provide feedback on the ﬁndings? | 7 |
| ***Reporting*** |  |  |
| **29. Quotations presented** | Were participant quotations presented to illustrate the themes/ﬁndings? Was each quotation identiﬁed? e.g. participant number | 8-15 |
| **30. Data and ﬁndings consistent** | Was there consistency between the data presented and the ﬁndings? | See discussion: 15-17 |
| **31. Clarity of major themes** | Were major themes clearly presented in the ﬁndings? | 8-15 |
| **32. Clarity of minor themes** | Is there a description of diverse cases or discussion of minor themes? | 15 |

**Supplementary material 2: Final interview schedule**

- There are many different words and terms often given for people who – for whatever reason does not drink alcohol or drinks very occasionally. Do you have a preferred term? (If they ask can give examples: teetotal, dry, sober, abstinent, or choosing not to drink, ‘non-drinker’, ‘light or occasional drinker’). Use the person’s chosen term for the rest of the interview.
- If you talk about anyone else during the interview by name (such as a friend or member of staff) – then we will anonymise this during the transcription process and provide them with a pseudonym. Likewise, you (the participant) will also be anonymised during the write-up and be given a false name in any write-up and reports resulting from this study
- Student to ask participant whether it would be helpful or not to them to know about her own alcohol consumption/drinking experiences, to help them feel comfortable.
  - If yes – provide brief explanation (we will discuss wording)
  - If no – proceed below

Beginning the interview

*Start recording the interview.*

**Rapport building**

Q. Would you be able to tell me the main reason for being a person who drinks little or no alcohol [or participant’s term]?

Q. Can you tell me if you arrived at university as a person who drinks little or alcohol [or participant’s term] or if you stopped/started drinking since being at university?

**Part 1. Arriving and settling into university (enquire about past, first three months)**

Q. Can you tell me about your experiences of settling into university in the first few months of first year?

*Follow up questions: Are there any differences in how settled you feel now?*

Q. Can you tell me about your experience of Fresher’s week when you first arrived at University being a low/non-drinker?

*Follow up questions: What events did you attend? Were there any events you wanted to attend but didn’t? Did you feel a certain pressure to attend?*

Q. Can you tell me about your experiences of meeting people in the first few months?

Q. Can you talk about your experiences of university accommodation when you first arrived? Who did you live with?

*Follow up questions: What was it like? Did you feel as though alcohol ever affect those first few days?*

**Part 2. Being around other drinkers**

Q. Can you talk about how you feel being around people who drink alcohol?

Q. Do you ever get asked why you don’t drink?

*Follow up: How do you feel about being asked that? Do you ever lie about why?*

Q. Have you ever felt the need to make excuses/or give specific reasons for not drinking?

*Examples: I’m taking medication, I’m allergic, I have drinking problem*

Q. Have you ever experienced pressure to drink?

*Follow up questions: By who? (ensure doesn’t mention specific names). Can you give an example of one of these situations?*

Q. Have you ever pretended to drink to fit in?

Examples, buying own drink with no alcohol, pouring away drinks if they were bought for you

**Part 3. Friendship and social life**

Q. Tell me about your social life at the moment (may be different now due to COVID-19).

Follow up: How was your social life at the start of university? Has it changed? If so, how?

Q. Can you talk about your main friendship group at university (rather than people at home)?

Q. Do you think being a low/non-drinker [or participant’s term] affected making new friends at university? If so how? If not, why?

Q. How many of your friends do not drink and how many do?

Follow up questions: did any friends previously not drink and now do? What do you think of this?

Q. Are you part of any societies at university, and do they involve drinking?

Finally, have you noticed any difference in people’s attitudes to you not drinking/drinking little alcohol over the years?

Follow up questions: For the better? For the worse? Why do you think that is?

Okay that reaches the end of the questions I wanted to ask you. Is there anything else you wanted to add or talk about that we didn’t talk about today?

If you’re okay to end the interview there, I’ll stop recording thank you!

**Debriefing**

- Thank you for the interview
- How are you feeling – is there anything in the interview has troubled you or distressed you?
- Can e-mail or send in the chat box on Microsoft Teams the participant with the debriefing sheet which outlines range of services etc, go through it with them. If there is any particular service/resource that they have expressed an interest in – then signpost them to it.
- If you don’t know how to answer a question they have – then you can forward what they’ve said onto me and I will respond to them.
- State to them that you will email them tomorrow just to say thank you again and to provide a PDF version of the sheet. They don’t need to respond to this, unless they’ve got anything else they want to add or want to request some additional information.
- Thank them again and log off/hang up

**Supplementary material 3: Amendments made to the pilot interviews before use.**

| Before Piloting | After Piloting |
| --- | --- |
| There were no questions about the potential peer pressure to attend events or to drink.  ‘Have you ever used any strategies to cope with being around other drinkers?’.  ‘Have you ever deceived others about your drinking?’  The opinions towards friends who do or do not drink were not discussed.  The changing attitudes of others towards the participants behaviour was not discussed. | A follow-up question to the experience of Fresher’s week was added; ‘did you feel a certain pressure to attend?’  The question, ‘have you ever experienced pressure to drink?’, was added in the subheading, ‘Being around other drinkers’, as it felt this was missing in interview.  Responses from pilot participants focused more on mental health strategies, which is discussed more broadly, rather than physical coping strategies. The question was changed to; ‘have you ever felt the need to make excuses/or give specific reasons for not drinking?’.  Pilot participants didn’t understand the word ‘deceived’ until EC had re-worded it or given examples. The question was changed to; ‘have you ever pretended to drink to fit in?’  A follow up question to ‘how many of your friends do not drink and how many do?’ was added; ‘did any friends previously not drink and now do? What do you think of this?’  Final question added; ‘have you noticed any difference in people’s attitudes to you not drinking/drinking little alcohol over the years?’. With follow-up questions; ‘for better? For worse? Why do you think that is?’. It was deemed important to look at how their experience has changed throughout university. |

**Supplementary Table 1. Social Provisions Scale (SPS-24) sub-scale results for each student**

| **Pseudonym** | **Reliable Alliance** | **Attachment** | **Nurturance** | **Social Integration** | **Reassurance of Worth** | **Guidance** |
| --- | --- | --- | --- | --- | --- | --- |
| Harry | 8 | 4 | 5 | 13 | 10 | 8 |
| Mia | 16 | 16 | 16 | 16 | 12 | 16 |
| Amir | 16 | 10 | 16 | 15 | 14 | 13 |
| Zoe | 15 | 16 | 12 | 15 | 12 | 15 |
| Aisha | 14 | 15 | 15 | 14 | 15 | 14 |
| Sophie | 16 | 11 | 8 | 13 | 12 | 13 |
| Iman | 16 | 14 | 15 | 16 | 16 | 16 |
| Emma | 16 | 16 | 14 | 16 | 15 | 16 |
| Olivia | 11 | 16 | 11 | 14 | 12 | 15 |
| Danielle | 13 | 14 | 15 | 10 | 14 | 10 |
| Amara | 16 | 13 | 12 | 16 | 15 | 16 |
| Lily | 14 | 10 | 8 | 12 | 12 | 13 |
| Grace | 16 | 14 | 15 | 14 | 16 | 16 |
| Kate | 15 | 13 | 14 | 14 | 12 | 14 |
| Jess | 15 | 16 | 12 | 14 | 13 | 15 |

Notes: The six-factor scores range from 4 to 16 which higher scores indicating better perceived support. Reliable alliance (‘there are people I can depend on to help me if I really need it’), attachment (‘I have close relationships that provide me with a sense of emotional security and well-being’), nurturance (‘there are people who depend on me for help’), social integration (‘There are people who enjoy the same activities that I do’), reassurance of worth (‘I have relationships where my competence and skill are recognised’), and guidance (‘there is someone I could talk to about important decisions in my life’).
