# Supplementary material for: “Drunk People Are on a Different Level”: A Qualitative Study of Reflections From Students About Transitioning and Adapting to United Kingdom University as a Person Who Drinks Little or No Alcohol
Source: Front Psychol. 2022 Jan 27;12:702662. doi: 10.3389/fpsyg.2021.702662 (PMC8829061; doi:10.3389/fpsyg.2021.702662)
Supplement: Supplementary file 2 [file Data_Sheet_2.docx]

**Supplementary figure 1. Flowchart of participants recruited in the study**


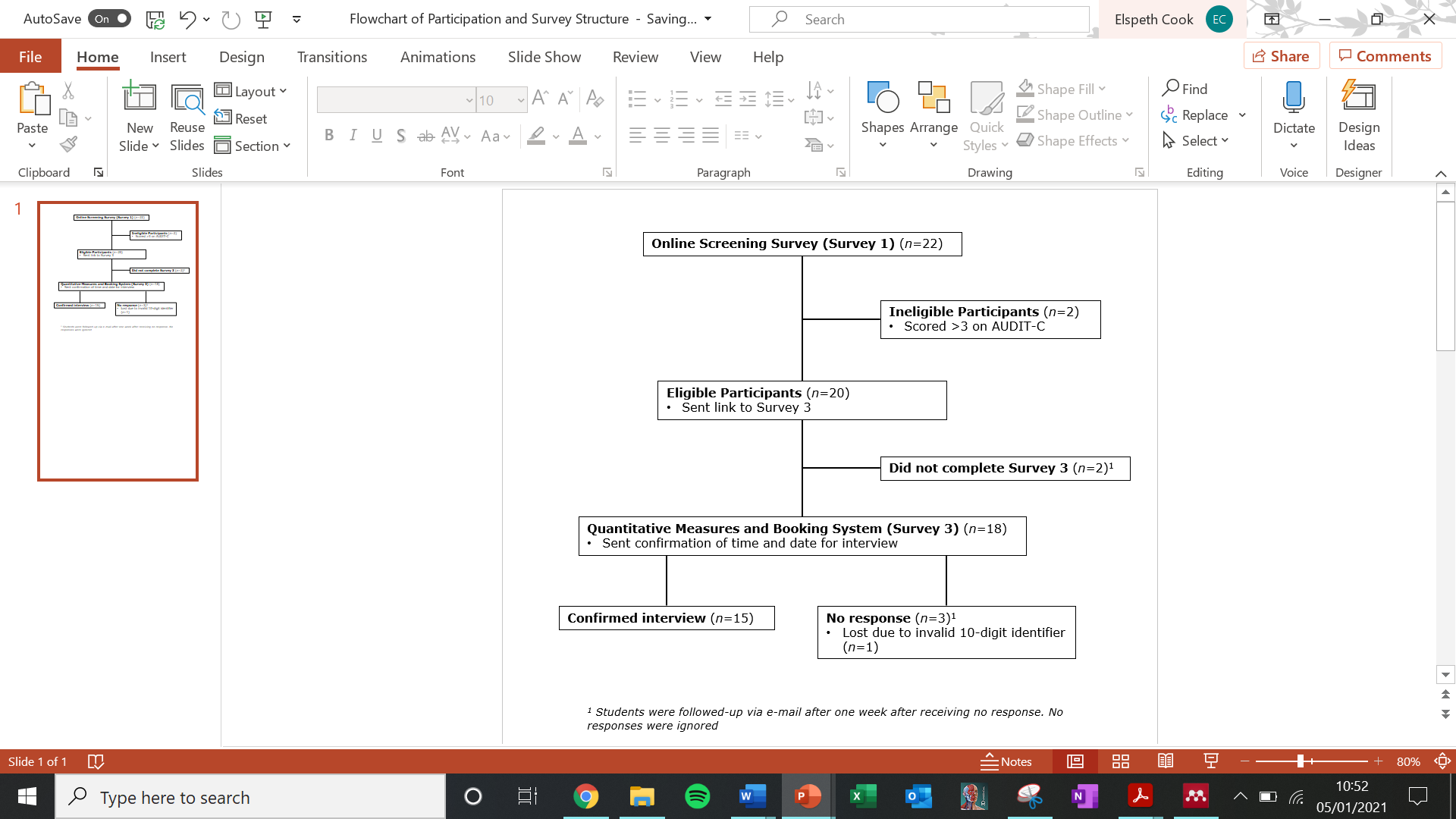


^1^ Students were followed up via e-mail one week after receiving no response. If a further no response, these students were not included in the study.
